# Functional Genomics in Health and Disease

**DOI:** 10.3390/ijms222312944

**Published:** 2021-11-30

**Authors:** Cornelia Braicu

**Affiliations:** Research Center for Functional Genomics, Biomedicine and Translational Medicine, Iuliu Hatieganu University of Medicine and Pharmacy, 23 Marinescu Street, 400337 Cluj-Napoca, Romania; cornelia.braicu@umfcluj.ro

Functional genomics applied in clinical disease diagnosis and prognosis allow the achievement of the progress in all aspects of biology in health and disease. This Special Issue of IJMS on the topic of “Functional Genomics in Health and Disease” has practical importance, trying to encompass the full biology of the coding and non-coding genes.

In this Issue, Maestri presents a Long-Read Sequencing Approach for Direct Haplotype Phasing in Clinical Settings. This has great translational significance, as analysis can be performed for multiple samples. These predictive biomarkers can be used for patient selection for treatment groups in clinical settings [1].

Vyse and colleagues cover the important topic of Trans-Ancestral Fine-Mapping and Epigenetic Annotation used as tool to Delineate Functionally Relevant Risk Alleles in Systemic Lupus Erythematosus [2].

Dudea-Simon and colleagues report the involvement of certain coding genes in cervical cancer, using an integrative analysis, and the diagnostic and prognostic potential of key core genes for this disease [3].

Biswas and colleagues review the role of a highly expressed miRNA pattern, and discuss the data related to its potential interaction of miRNAs with key signaling pathways in infected monocyte-derived macrophages and peripheral mononuclear blood, allowing the identification of unique biomarkers that can differentiate HIV-1 and HIV-2 infection based on an altered miRNA pattern [4].

Zayed present a novel bioinformatics method for the assessment of the molecular genetic susceptibility pattern in asthma. This paper led to the identification of novel genetic risk factors responsible for the development of moderate-to-severe asthma, serving as a platform for better treating asthma [5].

Potashkin et al. present bioinformatics analysis, which was focused on the key genes and molecular pathways that may trigger dementia. This study identified phosphodiesterase 4D-Interacting Protein as key factor for the frontal cortex dementia switch gene [6].

Chitoiu and colleagues discuss the roles of extracellular vesicle biology, a particularly important topic in light of the recent success of the multi-omics data [7].

The review by Jurj et al. gives an overview of the genome wide association studies, mainly in triple negative breast cancer, providing significant and new knowledge for both research and clinical studies [8].

Thus, the aim of this Special Issue is to answer to many of our readers’ questions regarding this topic, and we also hope that will open new avenues of research, providing important discoveries in this ongoing revolution.
